# H_2_O_2_ Production Downstream of FLT3 Is Mediated by p22phox in the Endoplasmic Reticulum and Is Required for STAT5 Signalling

**DOI:** 10.1371/journal.pone.0034050

**Published:** 2012-07-13

**Authors:** John F. Woolley, Ruth Naughton, Joanna Stanicka, David R. Gough, Lavinia Bhatt, Bryan C. Dickinson, Christopher J. Chang, Thomas G. Cotter

**Affiliations:** 1 Tumour Biology Laboratory, Biochemistry Department, Bioscience Research Institute, University College, Cork, Ireland; 2 Department of Chemistry, University of California, Berkeley, California, United States of America; 3 Howard Hughes Medical Institute, University of California, Berkeley, California, United States of America; Cincinnati Children's Hospital Medical Center, United States of America

## Abstract

The internal tandem duplication (ITD) of the juxtamembrane region of the FLT3 receptor has been associated with increased reactive oxygen species (ROS) generation in acute myeloid leukemia (AML). How this elevated level of ROS contributes to the leukemic phenotype, however, remains poorly understood. In this work we show that ROS in the FLT3-ITD expressing AML cell line MV4-11 is reduced by treatment with PKC412, an inhibitor of FLT3, DPI, a flavoprotein inhibitor, and VAS2870, a Nox specific inhibitor, suggesting that ROS production is both FLT3 and NADPH oxidase dependent. The majority of these ROS co-localize to the endoplasmic reticulum (ER), as determined with the H_2_O_2_-specific aryl-boronate dye Peroxyorange 1, which also corresponds to co-localization of p22phox. Moreover, knocking down p22phox dramatically reduces H_2_O_2_ after 24 hours in the ER, without affecting mitochondrial ROS. Significantly, the FLT3 inhibitor PKC412 reduces H_2_O_2_ in FLT3-ITD expressing cell lines (MV4-11, MOLM-13) through reduction of p22phox over 24 hours. Reduced p22phox is achieved by proteasomal degradation and is prevented upon GSK3-β inhibition. Knockdown of p22phox resulted in reduced STAT5 signalling and reduced Pim-1 levels in the cells after 24 hours. Thus, we have shown that FLT3 driven H_2_O_2_ production in AML cells is mediated by p22phox and is critical for STAT5 signalling.

## Introduction

Aberrant signalling through receptor tyrosine kinases (RTKs) is known to be a significant pathway in tumour development and perpetuation [1]. FMS-like tyrosine kinase 3 (FLT3) is a type III RTK expressed in approximately 90% of acute myeloid leukemia (AML) and activating mutations of FLT3 are found in approximately 30% of all AML cases [2]. In fact, FLT3 is the most frequently mutated gene associated with AML [3]. The most prevalent mutation of FLT3 is the internal tandem duplication (ITD) of the juxtamembrane domain conferring constitutive activation of the tyrosine kinase domain, leading to autophosphorylation of the receptor and subsequent phosphorylation of substrate proteins [2]. Moreover, several studies have demonstrated that the prognosis for AML patients with FLT3-ITD is relatively poor [4]. Also of significant clinical relevance is the association of FLT3-ITD with increased chemoresistance in AML patients [5]. Constitutive activation of FLT3 switches on downstream signalling pathways such as PI3K/Akt, Ras/Raf/MAPK and Jak/STAT [6]. Activation of these pathways in myeloid cells is known to promote survival, proliferation, and transformation [7], [8], [9]. Interestingly, FLT3-ITD expressing cell lines are known to have increased levels of endogenous reactive oxygen species (ROS) [10], although it is still unclear precisely what advantage this confers on the cells.

ROS were traditionally thought of as an unwanted by-product of cellular respiration, but in recent years there has been in a renaissance of research into their role as mediators of intracellular signalling [11]. Key to the renewal of interest in the field was that levels of endogenous ROS in some tumour cells are elevated [12], [13], [14]. Major oncogenes such as *Bcr/Abl*, *Ras* and *c-myc* have all been shown to induce ROS production [15], [16]. The increased level of steady-state ROS has been linked to numerous cellular processes associated with tumour development such as transformation, upregulation of pro-survival pathways or DNA-damage inducing mutations. The imbalance is derived either from an increase in ROS production or through a decrease in levels of ROS scavenging proteins [13]. Unravelling the precise mechanisms involved has proven to be difficult and to date we have a poor understanding of the precise role of ROS in tumours.

Similar to other tumours, increased ROS has been seen in a number of myeloid diseases. Haematopoietic growth factor signalling is mediated through ROS [17]. In T-cell acute lymphoblastic leukemia it has been shown that ROS regulates Phosphatase and tensin homolog (PTEN) contributing to increased viability in culture [18]. The development of Fanconi anemia has also been linked with elevated ROS driving genetic instability [19]. Work by our group and others demonstrated that in chronic myeloid leukemia (CML) Bcr-Abl induced ROS regulates the PI3K/Akt pathway [20], [21]. This work by Naughton *et* *al.* demonstrated that the NADPH oxidase Nox4 was responsible for producing ROS upon Bcr-Abl induction which contributed to survival in a myeloproliferative disorder (20).

**Figure 1:**
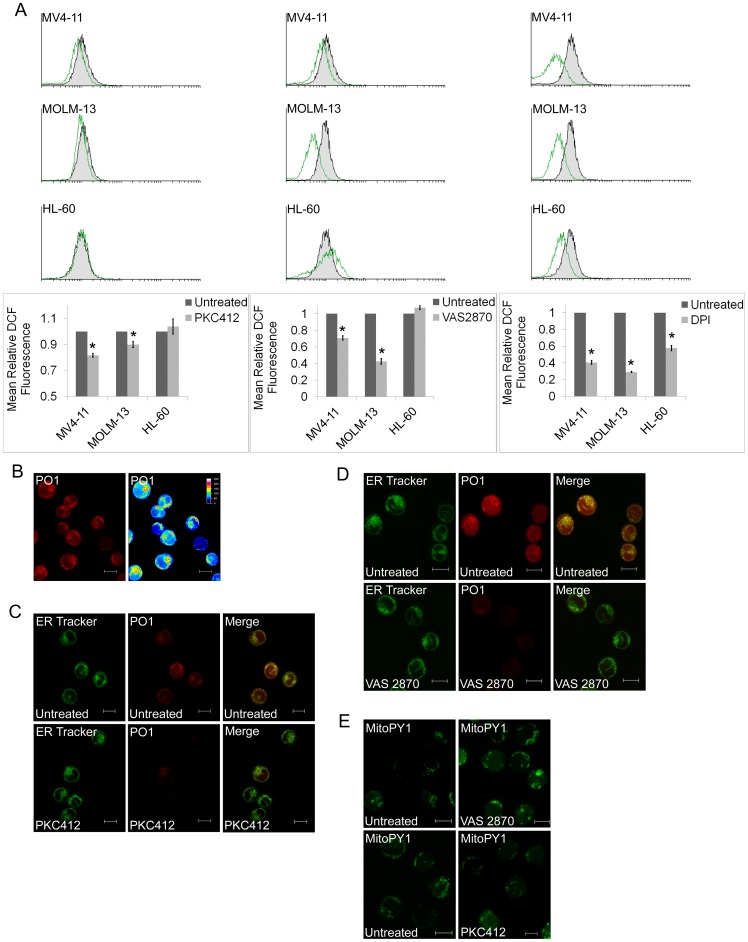
Inhibition of FLT3-ITD signalling reduces Nox-derived ROS in the Endoplasmic Reticulum. (a) Flow cytometric analysis of intracellular ROS as measured by DCF fluorescence in MV4-11, MOLM-13, HL-60 untreated leukemic cell lines (Unt) and following treatment with PKC412 (250nM) or VAS2870 (10μM) or DPI (5μM) for 1 hours. Mean fluorescence of untreated cells (black lines) is overlaid with that of PKC412 or DPI treated cells (green lines). Bar chart shows relative mean DCF fluorescence of PKC412, VAS2870 or DPI treated cells as a percentage of control untreated cells. All results are expressed as mean ± SD and are representative of three independent experiments. Statistical analysis was performed using ANOVA (*p<0.005). (b) MV4-11 cells were plated on the poly-D-lysine coated glass bottomed dishes and stained with Peroxyorange 1 (PO1). Pseudo-coloured image on the left represents intensity distribution (from highest intensity indicated by white to the lowest designated by black) (c) Cells were incubated with ER tracker, Peroxy Orange and where indicated with PKC412 (250nM;) or (d) VAS-2870 (10μM) for an hour at 37°C/5% CO_2_ followed by multi-tracking imaging using Zeiss LSM510 META confocal microscope. ER tracker and PO1 probes were excited at 488nm and 543nm respectively and their emissions were collected at 505–530nm and 560–615nm respectively (e) Cells were incubated with MitoPY1 and where indicated with PKC412 (250nM) or VAS-2870 (10μM) for an hour at 37°C/5% CO_2_ followed by imaging using Zeiss LSM510 META confocal microscope. MitoPY1 was excited at 514nm with a subsequent collection of emission between 505–550nm.

Nox proteins were originally identified in phagocytes, where Nox2 was shown to generate ROS as part of the innate immune system, but numerous homologues have since been identified outside of phagocytes [22]. These Nox proteins have been associated with signal transduction in a number of contexts and are now also associated with the development of numerous pathologies [23], [24]. Specific spatial and temporal regulation of Nox activity and/or expression confers a mechanism of controlling ROS within the cell and downstream pathways [25]. How the cell achieves this is not understood in much detail. In recent years a number of Nox regulator proteins have been uncovered but no clear overarching mechanism of regulation has been determined. Nox-derived ROS are known to play a role in FLT3-ITD AML, with increased production associated with the mutant receptor [10]. Sallmyr *et* *al.* also showed that this was associated with increased binding of Rac1 to Nox2, suggesting that Nox2 was at least partly involved.

**Figure 2:**
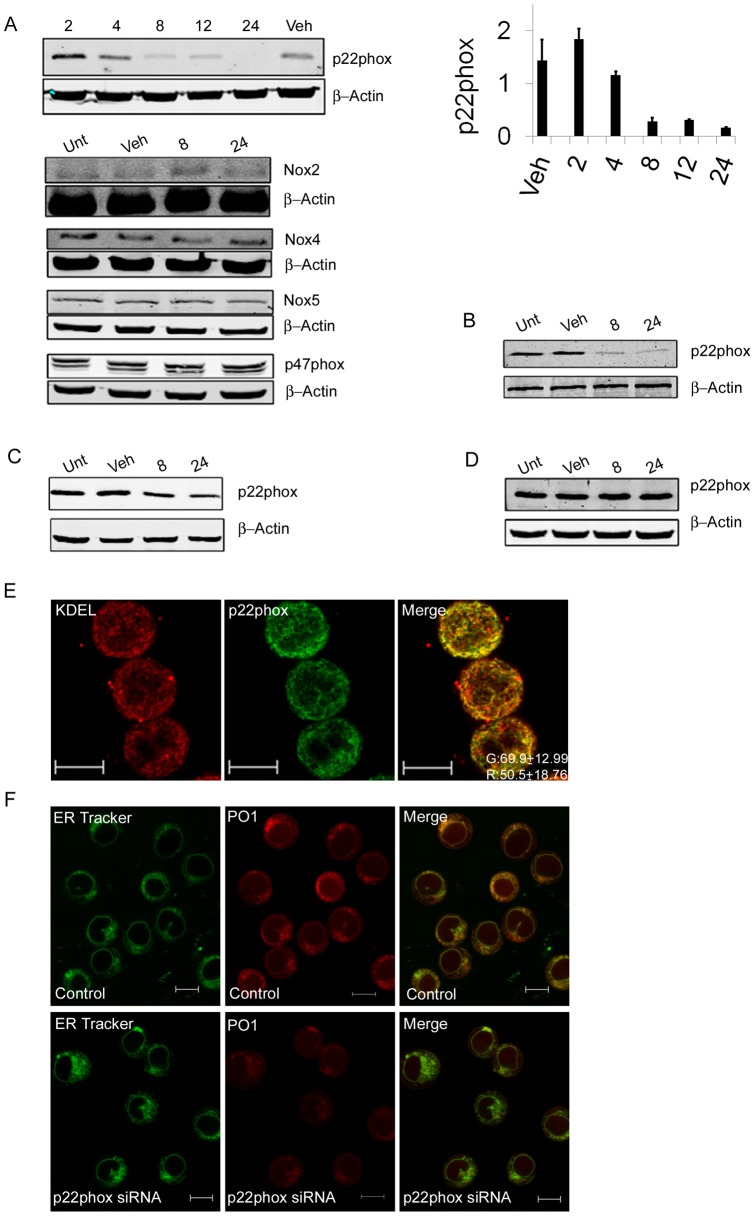
Inhibition of FLT3-ITD signalling reduces p22phox levels in the Endoplasmic Reticulum. (a) Western blot analysis of Nox protein expression in untreated MV4-11 cells (Unt), vehicle controls (Veh) and following treatment with PKC412 (250nM) over 24 hours using β-Actin as loading control. Histograms represent the ratio of the intensity of target bands quantified by densitometry factored by the densitometric measurement of loading control band. The data are expressed as percentage of control, where the ratio in the control was defined as 1. Values are the mean ± SD and are representative of three independent experiments. Western blot analysis of p22phox protein expression: (b) in untreated MOLM-13 (Unt), vehicle controls (Veh) and following treatment with PKC412 (250nM); (c) in untreated MV4-11(Unt), vehicle controls (Veh) and following treatment with CEP-701 (50nM); (d) in untreated HL-60 (Unt), vehicle controls (Veh) and following treatment with PKC412 (250nM) over 24 hours using β-Actin as loading control.(e) MV4-11 cells were cultured for 16 hours in Poly-D-lysine coated glass bottomed dishes. Cells were fixed in 3% PFA/PBS prior to staining with KDEL and P22phox antibodies. Images are represented as a single slice from a Z stack projection. Brightness and contrast adjustments have been made for convenience, with identical parameters applied across images. Relative measures of co-localization are included, values are the mean ± SD and are representative of three independent experiments. Digital images were analyzed with Meta-Morph software. Scale bar represents 10 μm. (f) Silencing p22phox reduces ROS generation. MV4-11cells were electroporated with either control siRNA or p22phox siRNA and plated in Poly-D-lysine coated glass bottomed dishes and stained with peroxyorange 1 (PO1), then imaged with a confocal laser scanning microscope. The same plates were then fixed and stained with the p22phox antibody for immunofluorescence. Brightness and contrast adjustments have been made for convenience, with identical parameters applied across images. The scale bar represents 10µm.

In this study we used a number of leukemic cell lines, expressing wild-type and mutated FLT3, to investigate elevated endogenous-ROS associated with FLT3-ITD. We show that the vast majority of H_2_O_2_ in these cells localize to the enodoplasmic reticulum (ER). Inhibition of FLT3 signalling by means of PKC412, which is currently in phase II clinical trials for AML [26], leads to a loss in this ER localized ROS concomitantly with the post-translational downregulation of the small membrane-bound component of the Nox complex, p22phox. Thus, we propose that increased H_2_O_2_ signalling via FLT3 in AML cell lines is derived from the ER via p22phox and that treatment of cells with PKC412 drives down ROS through regulation of p22phox.

**Figure 3:**
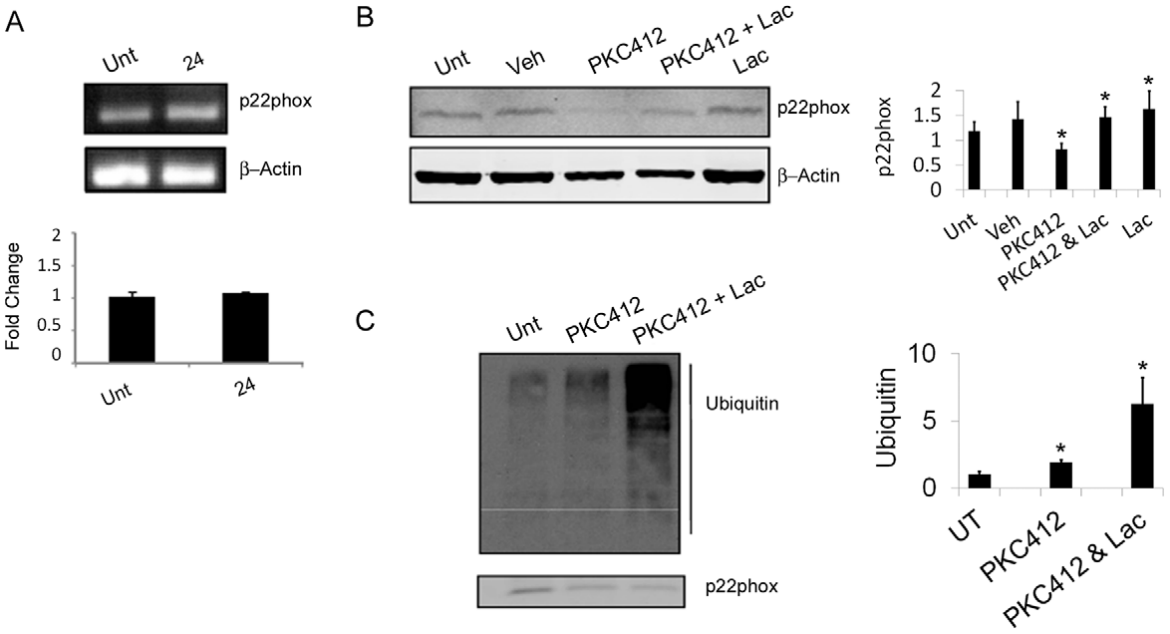
FLT3-ITD Inhibition results in proteasomal degradation of p22phox. (a) Products of quantitative PCR for p22phox mRNA in untreated MV4-11 cells (Unt) and cells treated with PKC412 (250nM) for 24 hours were visualised by agarose gel separation, loading control is β-Actin. Histogram shows relative expression of p22phox mRNA in untreated MV4-11 cells and cells treated with PKC412 (250nM) for 24 hours as determined by quantitative PCR ΔΔCt-method. Results are expressed as mean ± SD and are representative of three independent experiments. Statistical analysis was performed using student t-test (*p<0.005) (b) Western blot analysis of p22phox protein expression in untreated MV4-11 cells (UT), vehicle controls (Veh), and cells treated with PKC412 (250nM) and lactacystin (5μM; Lac) for 8 hours using β-Actin as a loading control. Histograms represent the ratio of the intensity of target bands quantified by densitometry factored by the densitometric measurement of loading control band. The data are expressed as percentage of control, where the ratio in the control was defined as 1. Values are the mean ± SD and are representative of three independent experiments (c) Immunoprecipitation of p22phox from whole-cell lysates of untreated MV4-11 cells (Unt), cells treated with PKC412 (250nM) and lactacystin (5 μM; Lac) for 8 hours. Nitrocellulose membranes were probed for the presence of ubiquitin. Values are the mean ± SD and are representative of three independent experiments.

## Materials and Methods

### Cell lines, culture conditions and treatment

The human leukemic cell lines MV4-11 and MOLM-13, (homozygous and heterozygous for the FLT3-ITD mutation, respectively) as well as HL-60, were maintained in RPMI 1640 medium supplemented with 10% fetal calf serum, 2mM L-glutamine and 1% penicillin/streptomycin (all from Sigma Aldrich, Dublin, Ireland) in a humidified incubator at 37°C with 5% CO_2_. Inhibition of FLT3-ITD signalling was achieved using PKC412 (250nM; Tocris Biosciences, Bristol, UK) for up to 24 hours. Nox inhibition was via flavoprotein inhibitor diphenyleneiodonium (5μM DPI; Sigma) or VAS2870 (10 μM; Enzo Life Sciences, Exeter, UK) for up to 1 hour. Inhibition of the 20S proteasome was via lactacystin (5μM; Sigma) for up to 8 hours. GSK3-β inhibition was via lithium chloride (10μM; Sigma) or SB 216763 (10μM; Tocris) for up to 8 hours.

**Figure 4:**
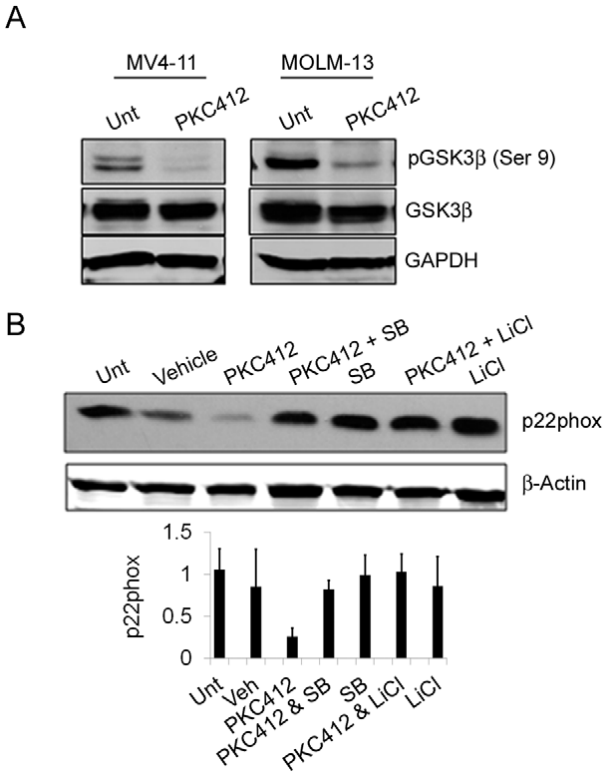
PKC412-mediated p22phox downregulation requires GSK3-β activation. (a) Western blot analysis of GSK3-β signalling in untreated MV4-11 and MOLM-13 cells (Unt), vehicle controls (Veh), and after treatment with PKC412 (250nM) for 16 hours or DPI (5μM) for 1 hour with GAPDH as a loading control (b) Western blot analysis of p22phox expression in untreated MV4-11 cells (Unt) and upon treatment with PKC412 (250nM), SB 216763 (10μM; SB), Lithium Chloride (10μM; LiCl) for 8 hours using β-Actin as a loading control. Histograms represent the ratio of the intensity of target bands quantified by densitometry factored by the densitometric measurement of loading control band. The data are expressed as percentage of control, where the ratio in the control was defined as 1. Values are the mean ± SD and are representative of three independent experiments.

### Measurement of Intracellular ROS by Flow Cytometry

Following treatments, ROS levels were determined using the cell-permeable fluorogenic probe 2, 7-dichlorodihydrofluorescin diacetate (DCF, Invitrogen Biosciences, Dun Laoghaire, Ireland). Briefly, DCF (50μM) was added to cells in suspension 30 min, and incubated in the dark, before analysis on a FACScalibur (Becton and Dickinson, Oxford, UK) with excitation and emission spectra set at 488 and 530 nm, and using CellQuest software. H_2_O_2_ and O_2_^−^ production was calculated by the increase in mean fluorescence.

**Figure 5:**
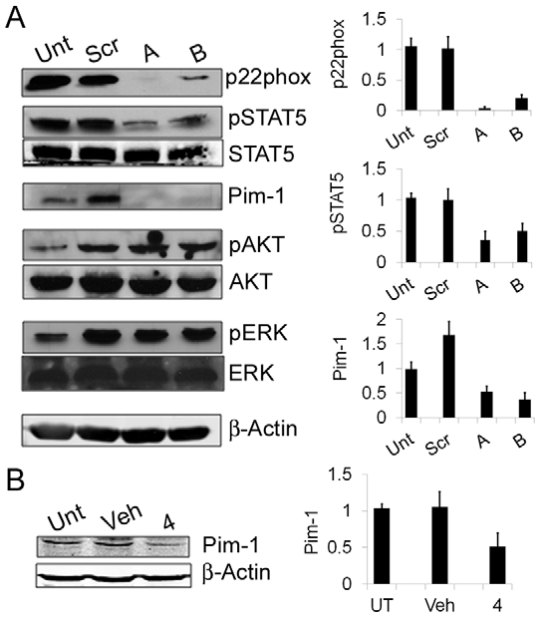
p22phox links FLT3-ITD to STAT5 signalling in AML. (a) Western blot analysis of signalling pathways in untreated MV4-11 cells (Unt) and 24 hours post knockdown of p22phox protein levels via siRNA (A, B). Cells were transfected with either of two siRNA oligomers for p22phox knockdown or a negative control oligomer (Scr) and compared to untreated cells 24 hours post-transfection. Histograms represent the ratio of the intensity of target bands quantified by densitometry factored by the densitometric measurement of loading control band. The data are expressed as percentage of control, where the ratio in the control was defined as 1. Values are the mean ± SD and are representative of three independent experiments (b) Western blot analysis of Pim-1 protein expression in untreated MV4-11 cells (Unt), vehicle controls (Veh) and following treatment with VAS2870 (10μM) over 4 hours using β-Actin as loading control. Histograms represent the ratio of the intensity of target bands quantified by densitometry factored by the densitometric measurement of loading control band. The data are expressed as percentage of control, where the ratio in the control was defined as 1. Values are the mean ± SD and are representative of three independent experiments.

### Antibodies

Primary antibodies used for immonoblotting or immunoprecipitation were Akt (#9272), phospho-Akt (Thr308; #9276S), ERK (#9102), phospho-ERK (Thr202/204; #9275S), GSK3-β (#9315), phospho- GSK3-β (Ser9; #9336S), Pim-1 (#2907S; all from Cell Signalling Technology, Boston, MA, USA), Nox1 (#SC25545), Duox (sc-48858), p22phox (#SC20781; all from Santa Cruz Biotechnology, Santa Cruz, CA, USA), GAPDH (#RGM2-500; Advanced Immunochemicals, Long Beach, CA, USA), β-Actin (#A5441; Sigma), STAT5 (#610191; BD Biosciences, Oxford, UK), phospho-STAT5 (Tyr694/699; #04-886), ubiquitin (#MAB1510), Nox2 (#07-024; all from Millipore, Cork, Ireland), KDEL (#ab12223; Abcam, Cambridge, UK). Nox4 antibody was a kind gift from Dr JD Lambeth (Emory University School of Medicine, Atlanta, GA, USA). Nox5 antibody was a kind gift from Dr WM Nauseef (Department of Medicine, University of Iowa and the Veterans' Administration Medical Center, Iowa City, IA, USA). All secondary antibodies for western blotting were peroxidase conjugated (Dako, Glostrup, Denmark).

**Figure 6:**
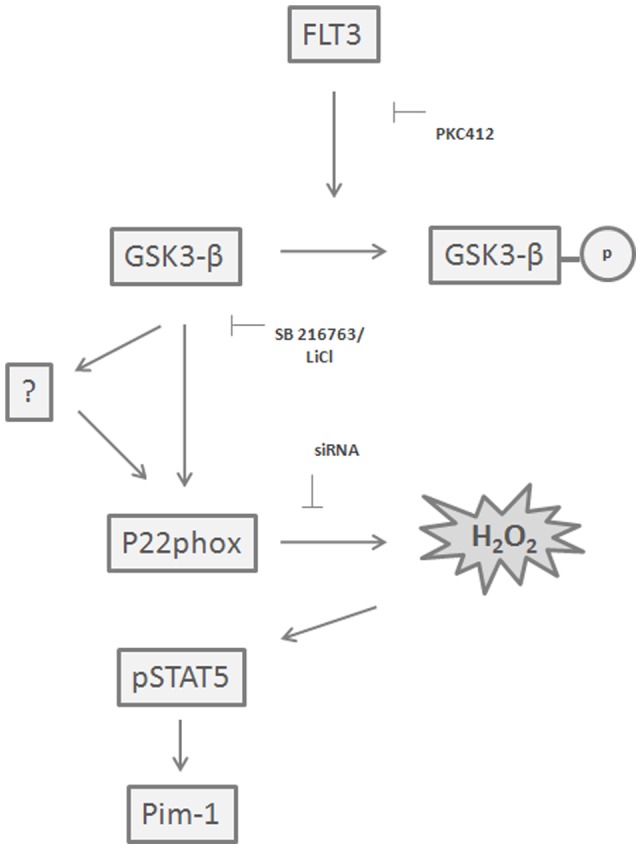
Schematic of Proposed Mechanism for p22phox-mediated H_2_O_2_ signalling in FLT3-ITD AML cell lines. p22phox is required for the production of H_2_O_2_ in AML cell lines at the Endoplasmic reticulum. Inhibition of FLT3 signalling in these cells leads to degradation of p22phox by the proteasome, which requires active GSK3-β. Removal of p22phox from this system halts STAT5 signalling to Pim-1.

### Immunofluorescence and Microscopy

Confocal fluorescence live imaging studies were performed with a Zeiss LSM510 META confocal microscope fitted with a 63×1.4 plan apochromat lens. Excitation of PO1 at 543nm was carried out with Ar laser and emission was collected between 560–615nm. Excitation of MitoPY11 at 514nm was carried out using Ar laser and collected between 505–550nm. Excitation of ER tracker blue at 405nm was carried out using HeNe laser and emission was collected between 420–480nm. Excitation of ER tracker green at 488nm was carried out using Ar laser and emission was collected between 505–530nm. The multi-tracking mode of scanning was applied for acquisition of the images. Image analysis was performed in MetaMorph Offline and Carl Zeiss Zen 2009 Light Edition.

Aprroximately 4–5 hours (for PKC412 and VAS-2870 treatments) or 24 hrs (after siRNA transfection) before imaging MV-411 cells were plated on poly-D-lysine (#P4707; Sigma) coated glass bottomed dishes (#P35G-1.5-14-C; MatTek Corporation, Ashland,). 1hr before imaging, ER tracker dye (1µM; Molecular probes), Peroxy Orange (PO1; 15µM), Mito PY1 (8µM) were added. Where indicated, cells were treated with PKC412 (200nM) or VAS-2870 (10µM) for 1hr before imaging. After treatment, cells were washed twice with PBS buffer and incubated in fresh medium during imaging.

Briefly, for siRNA experiments MV4-11 cells were cultured for 24 hours before the experiment in Poly-D-lysine coated glass bottomed dishes and imaged as previously described [27]. For localization studies of p22phox, MV4-11 cells were cultured for 16 hours on Poly-D-lysine coverslips then fixed for 1hr in 3% PFA/PBS. The cells were then permeabilised using 0.05% saponin/0.2% BSA/PBS for 5 min. The coverslips were incubated with 50µl of KDEL and p22phox primary antibody solutions (1/100 in FBS/PBS) and incubated for 1hr following washing with PBS. 50µl of Alexa fluor 594 and Alexa fluor 488 secondary antibody solutions (1/200 in FBS/PBS) were added onto coverslips and incubated for 1hr. The coverslips were washed with PBS and water and mounted on the slides using moviol. Images were acquired using multiphoton laser scanning microscope Flouview1000 MPE (Mason Technology Dublin, Ireland) with 100x oil immersion objective. Images are represented as a single slice from a Z stack projection. The p22phox/Alexa fluor 488 antibody was excited using 488nm wavelength and KDEL/Alexa fluor 594 was excited using 543nm wavelength. The sequential scanning mode was applied for the acquisition of the images. Digital images were analyzed with Meta-Morph software (Universal Imaging, West Chester, PA).

### Immunoblotting

Cells were lysed with RIPA buffer [Tris–HCl (50mM; pH 7.4), 1% NP-40, 0.25% sodium deoxycholate, NaCl (150mM), EGTA (1mM), sodium orthovanadate (1mM), sodium fluoride (1mM), cocktail protease inhibitors (Roche, Welwyn, Hertforshire, UK) and 4-(2-Aminoethyl) benzenesulfonyl fluoride hydrochloride (200mM)] for 20 min on ice followed by centrifugation at 14 000 g for 15 min to remove cell debris. Equivalent amounts of protein, as determined by the Bio-Rad Protein Assay (Bio-Rad, Hemel Hempstead, UK) were resolved using SDS–polyacrylamide gel electrophoresis followed by transfer to nitrocellulose membrane (Schleicher and Schuell, Dassel, Germany) and incubated overnight with the appropriate antibodies. Membrane development was achieved using enhanced chemiluminescence (GE Healthcare, Buckinghamshire, UK).

### Immunoprecipitation

Briefly, approximately 1×10^7^ cells were lysed, as described above, and reconstituted at a concentrated of 1mg/mL with RIPA buffer. Total protein was incubated with anti-p22phox antibody (1/100 dilution) for 2 hours with rocking at 4°C. 20μL of Protein A/G PLUS –Agarose (Santa Cruz Biotechnology) was then added and incubated overnight at 4°C. This solution was then spun down at maximum speed in a microcentrifuge for 1 minute and the supernatant was removed. The beads were washed 5 times with PBS prior to resuspension in loading dye with Dithiothreitol (DTT; Sigma) and boiling at 90°C for 5 mins. This solution was then spun down at maximum speed in a microcentrifuge for 1 minute and the total supernatant resolved using SDS–polyacrylamide gel electrophoresis, as described above.

### Small Interfering RNA (siRNA)

RNA interference mediated by duplexes of 21-nucleotide RNA was performed in MV4-11 cells. Different Ambion Silencer Select predesigned siRNA (Applied Biosystems, Warrington, UK) were used for silencing. For p22phox, the siRNA used were siRNA ID s3786 and s194371 (Ambion). For the negative control, the siRNA used were Silencer Select Negative Control #1 siRNA (Ambion). The sequences are available from the manufacturer website. The transfection of siRNA used the Amaxa Nucleofactor technology with the Amaxa cell optimization kit L (Amaxa, Cologne, Germany) and followed the Amaxa guidelines using program Q-001.

### Quantitative PCR

Quantitative PCR was performed on oligo-dT generated cDNA using the MJ Research Opticon 2 detection system in combination with the Quantitect SYBR Green PCR Master Mix (Qiagen, Crawley, UK). The primers for p22phox and β-Actin were purchased as Quantitect Primer Assays (Qiagen). The following PCR parameters were used for each primer set: denaturing at 95°C for 15 min, followed by 45 cycles of 94°C for 15 seconds, annealing temperature of 56°C for 30 seconds and extension at 72°C for 30 seconds. RNA sample was analyzed in triplicate, and p22phox expression relative to β-2-microglobulin was obtained by the equation &2circ;(Ct-Control)/&2circ;(Ct-Target). Data were represented as the mean relative expression ± standard deviation (SD). Statistical significance was evaluated by Student's t-test for comparisons between groups. PCR products were then visualised by separation on a 2% agarose gel and staining with SYBR Safe gel stain (Invitrogen).

### Statistical analysis

Data are given as mean ± SD. Statistical significance was evaluated by Student's t-test for comparisons between groups, and analysis of variance (ANOVA).

## Results

### Inhibition of FLT3-ITD signalling reduces Nox-derived ROS in the Endoplasmic Reticulum

MV4-11 and MOLM-13 are established IL-3 independent myelomonocytic leukemia cell lines homozygous and heterozygous for FLT3-ITD, respectively. Both of these cell lines have been used as models of FLT3-ITD AML in numerous studies to date and display a higher endogenous ROS level compared to cells with non-mutated receptors [10]. The receptor tyrosine kinase inhibitor PKC412 has been shown to inhibit autophosphorylation of mutant FLT3 receptors [28] and disrupt downstream signalling in AML cells [10]. We show that treatment of MV4-11 and MOLM-13 cells with PKC412 resulted in decreased endogenous ROS, and this decrease in ROS was not observed when either HL-60 cells were treated with PKC412 (Figure 1a). The level of ROS reduction in MOLM-13 and MV4-11 was comparable. Previous work, by our group and others, has linked Nox proteins with the production of ROS in leukemic cell lines [10], [21], [29]. Here, we show that treatment with DPI, a flavoprotein inhibitor, reduces the ROS in MV4-11 and MOLM -13 cells to a greater extent than in HL-60 (Figure 1a). This result is confirmed with the Nox-specific inhibitor VAS2870, which reduced ROS in the MV4-11 and MOLM -13 to a level comparable with FLT3-inhibition while no reduction of ROS was seen in HL-60 cells treated with VAS2870 (Figure 1a). Taken together these results suggest that Nox proteins are involved in the production of ROS downstream of FLT3-ITD signalling.

Recently FLT3 was shown to affect signalling outcomes in the endoplasmic reticulum (ER) [6], [30]. To investigate whether the increased ROS levels in FLT3-ITD cells is associated with this signalling we examined intracellular Peroxy Orange 1 (PO1) fluorescence using multi-photon microscopy in MV4–11 cells. We demonstrate that the highest level of ROS intensity seen with false colour imaging and the PO1 dye displays a consistent pattern (Figure 1b). Detection of endogenous H_2_O_2_ with PO1 correlates well with the ER-tracker dye suggesting an ER source for these ROS (Figure 1c & d). Importantly H_2_O_2_ in the ER was dramatically reduced by treatment with PKC412 and VAS2870 (Figure 1c & d; Figure S1), demonstrating that they are both FLT3-ITD and Nox dependent. To test whether treatment of MV4-11 cells with these inhibitors had any effect on mitochondrial sources of ROS we incubated them with the MitoPY1 dye (Figure 1e; Figure S2) [31]. Inhibiting either FLT3 or Nox proteins resulted in no reduction in mitchondrial ROS. Thus, these data demonstrate that FLT3 drives endogenous ROS in the ER through Nox proteins.

### Inhibition of FLT3-ITD signalling reduces p22phox levels in the Endoplasmic Reticulum

Given that both DPI and PKC412 treatment result in a large drop in ROS, we investigated whether Nox proteins changed in expression upon FLT3 inhibition, to determine if this reduction was mediated through a decrease in Nox protein expression (Figure 2a). We examined Nox proteins known to be expressed in leukemic cells [29]. While none of the Nox proteins themselves show a change in expression, we observed a reduction in the protein levels of the small membrane component of the Nox complex, p22phox, in MV4-11 cells (Figure 2a). p22phox was shown to decrease at 8 hours post PKC412 treatment, and none remains after 24 hours. A similar decrease at 8 and 24 hours was observed in the MOLM -13 cell line also (Figure 2b). To ensure this was a specific effect of the small-molecule inhibitor on FLT3-ITD signalling we treated the cells with CEP-701, another small molecule inhibitor of FLT3, and observed the same result (Figure 2c). Also we have seen that PKC412 had no effect on ROS or p22phox protein levels in cell lines not expressing the FLT3-ITD, namely HL-60 (Figure 2d). We then investigated whether the reduction in ROS seen upon PKC412 treatment was due to this downregulation of p22phox by examining the intracellular localization of the p22phox in MV4-11 cells. We show that p22phox co-localizes to the ER and its expression pattern mirrors that of the ROS as seen with PO1 (Figure 2e). Indeed our data show that approximately 70% of p22phox co-localizes with KDEL, while approximately 50% of KDEL co-localizes with p22phox. These data strongly suggest that p22phox is involved in the ER localized H_2_O_2_ production, but for further confirmation we selectively knocked down p22phox via siRNA. Cells in which p22phox was knocked down were then compared to cells transfected with negative control siRNA, for ROS and p22phox localization. The knockdown resulted in an almost complete loss of p22phox protein in the cell and was accompanied by a dramatic drop in ROS levels in the ER (Figure 2f; Figure S3) similar to treatment with PKC412. These results indicate that in MV4-11 cells that endogenous ROS production requires p22phox and localizes to the ER.

### FLT3-ITD Inhibition results in proteasomal degradation of p22phox

Given that p22phox protein levels are downregulated relatively rapidly post-treatment with PKC412 (Figure 2a), we investigated whether this effect was mediated post-transcriptionally. However, p22phox mRNA levels did not change significantly upon inhibition of FLT3-ITD up to 24 hours when the protein is substantially reduced (Figure 3a). Thus, it appeared likely that p22phox is regulated post-translationally. To test this hypothesis, we inhibited FLT3-ITD signalling in the presence of lactacystin, an inhibitor of the proteasome (Figure 3b). By blocking proteasome function we were able to partially abrogate the degradation of p22phox after PKC412 treatment. Thus, proteasomal degradation of p22phox occurs upon inhibition of FLT3 signalling. In order to elucidate how p22phox is targeted to the proteasome we immunoprecipitated the protein and probed for ubiqutin moieties as well immunoprecipitating ubiquitinated proteins and probing for p22phox (Figure 3c; Figure S4). We found that p22phox ubiquitination increases upon PKC412 treatment, and inhibiting the proteasome causes a build up of ubiquitinated p22phox in the cell indicating that p22phox is first ubiquitinated and then degraded by the proteasome. Taken together these data suggest that regulation via PKC412 was at the post-translational level.

### PKC412-mediated p22phox downregulation requires GSK3-β activation

Mutated FLT3 signalling is associated with constitutively activated signalling pathways [32]. Of particular interest is GSK3-β signalling, given its role in the regulation of proteasomal degradation in numerous contexts (32–34). GSK3-β is active when dephosphorylated and thus is likely to be redox regulated. We show that PKC412 inhibition of FLT3-ITD signalling prevents phosphorylation of GSK3-β in both MV4-11and MOLM-13 cell lines (Figure 4a). Given the known role for GSK3-β in proteasomal regulation we tested whether GSK3-β might be involved in the downregulation of p22phox seen here. Utilizing either of two known GSK3-β inhibitors, namely lithium chloride and SB 216763, we could almost completely reverse the degradation of p22phox after treatment with PKC412 (Figure 4b). Inhibiting AKT with LY 294002 had no effect on p22phox levels after PKC412 treatment (data not shown). These results indicate that GSK3-β facilitates the post-translational regulation of p22phox upon its activation by PKC412 dephosphorylation.

### p22phox links FLT3-ITD to STAT5 signalling in AML

It is known that one of the major pathways activated downstream of FLT3 in AML cells is STAT5, whose constitutive activation is associated with protection from apoptosis in mutant FLT3 cells [33]. STAT5 signalling downstream of mutant FLT3 has also been implicated in ROS generation [10]. Growth factor derived ROS can also mediate phosphorylation of STAT5 in leukemic cells [17], highlighting the dynamic role ROS can play in signalling pathways. We examined whether p22phox plays a role upstream of STAT5 in MV4-11 cells. Knockdown of p22phox mediated by siRNA gave a reduction in phosphorylation of STAT5 at 48hours (Figure 5a). Importantly, this was not accompanied by a reduction in Akt- or ERK-phosphorylation, which is in agreement with current understanding of FLT3-ITD signal transduction in AML cell lines [29]. This indicates that the reduction in phosphorylated-STAT5 was not due to a change in redox-state of the cells. Interestingly this was accompanied by a reduction in the levels of the serine/threonine kinase Pim-1, a target gene of STAT5. Pim-1 has recently been shown as a downstream effector of signalling by mutated FLT3 on the ER [6] and is known to regulate critical pathways in AML [20], [34]. A decrease in Pim-1 expression was also seen upon Nox-protein inhibition by VAS 2870 (Figure 5b) suggesting its regulation upon p22phox knockdown is through a reduction in Nox-activity also and is a downstream target of Nox signalling.

## Discussion

A number of studies have examined the role of ROS, and specifically Nox-derived ROS, in leukemia [10], [21], [29] but to date an understanding of how ROS contributes to a tumourous phenotype remains largely unknown. While previously considered an unwanted by-product of respiration, ROS contribution to intracellular signalling is now considered to be essential in healthy as well as tumourous cells. In this work we demonstrated for the first time that FLT3-ITD drives H_2_O_2_ production in the ER and that this is mediated by the small subunit of the NADPH oxidase, p22phox. Also, we have shown that p22phox signalling affects mediators known to be critical for leukemic cell development and survival.

Nox proteins have been seen localized to the ER in various cell line types [35], [36], [37]. However, to date no evidence for Nox-derived ROS signalling localized to the ER has been presented in AML. Also, here we show live-cell imaging of localized ROS in leukemia cells using PO1 fluorescence. While it is difficult to quantify ROS based on DCF fluorescence [38], [39], we show that H_2_O_2_ detected by the highly-specific PO1 dye localizes to the ER. This dye has been shown to detect endogenous levels of hydrogen peroxide [40] and here we demonstrate it can be used for co-localization studies, showing that ROS generated in MV4-11 cells co-localise with ER tracker dyes. Thus, the H_2_O_2_ generated downstream of FLT3 appears to be H_2_O_2_, the highest concentration of which is in the ER. Recently, it was shown that Nox-derived ROS induced by oxidized low-density lipoprotein (OxLDL) in monocyte-derived macrophages localized to the ER [41]. Our localization of H_2_O_2_to the ER is similar to that of Lee *et al.,* in an analogous system of suspension myeloid cells [41]. The ROS generation we see is almost completely inhibitable by both PKC412 and DPI, indicating that it is both FLT3- and Nox-dependent (Figure 1). Signalling downstream of the FLT3-ITD results in a number of pathway outcomes, and of particular interest is the activation of STAT5 at the ER [6], [30]. This compartmentalization of signalling processes downstream of FLT3-ITD correlates with the ROS localization we have shown here. Nox proteins are generally membrane-bound and although there is evidence for Nox localization throughout the cell these results are nonetheless surprising by suggesting that the Nox complex in AML generates significant amounts of ROS at the ER. Some evidence suggests that Nox proteins assemble with p22phox at the ER prior to the mature heterodimer complex trafficking to the plasma membrane [42], however in our system ROS are generated at the ER.

Nox family members have been associated with numerous signalling pathways relevant to survival, apoptosis, migration and transformation in AML cells [43] all of which make them excellent candidates for study. Here we have shown that inhibition of FLT3-ITD signalling results in the downregulation of p22phox (Figure 2a) without affecting the expression of other Nox proteins. p22phox was first identified as the small membrane-associated subunit of flavocytochrome b_558_ [44] and has since been identified as as a component of Nox1-4 [45]. Full functioning of these Nox proteins require p22phox [46], [47], [48]. Thus, the complete loss of p22phox in the cell is likely to have significant effects on any ROS regulated pathways. Also, recent evidence shows that p22phox localizes with Nox4 in the ER in human monocyte-derived macrophages [41] suggesting that loss of p22phox at the ER in our system could regulate ROS derived from Nox4. Here we show for the first time that p22phox can be found in the ER in AML cell lines and indeed appears to be required for the high levels of ROS found there (Figure 2e). Therefore, it appears that inhibition of FLT3 signalling leads to a downregulation of p22phox and this in turn drives down the level of ROS in the cell dramatically.

Given that the levels of p22phox drop relatively soon after FLT3 inhibition, the question was raised as to how this was mediated. We have shown that the level of mRNA is unaffected by PKC412 and that proteasomal inhibition prevents protein degradation after ubiquitination. To our knowledge, treatment with PKC412 has not been reported to induce the proteasomal degradation of any proteins in AML cells and that this occurs to p22phox is a significant finding. Confirming that this was not a general stress response due to removal of FLT3 signalling we have seen that chaperone proteins Hsp70 and Hsp90, as well as a marker of the unfolded-stress response Grp78, are all unaffected by PKC412 treatment (data not shown). Interestingly, it is known that the von Hippel-Lindau tumor suppressor gene (VHL) is responsible for downregulating p22phox in renal carcinoma cells via ubiquitination and subsequent proteasomal degradation [49]. Thus, p22phox is downregulated by the tumour suppressor gene VHL and by inactivation of the oncogene FLT3. This raises the intriguing possibility that p22phox plays a role linking oncogenesis and downstream effectors in a more general context and that it is not simply involved in these particular disease models.

Inhibition of FLT3 signalling was associated with an increase in activated GSK3-β, through a reduction in its deactivated phosphorylated-form. We have demonstrated that this activation of GSK3-β is essential for the reduction of p22phox levels (Figure 4). Inhibition of GSK3-β completely abrogates p22phox protein degradation, which demonstrates that without GSK3-β signalling p22phox cannot be processed by the proteasome. GSK3-β is known to target proteins to the proteasome with P21, Cyclin D3 and HIF-1α, for example, all processed in this manner [50], [51], [52]. While it is known that p22phox can be processed to the proteasome via ubiquitination, a role for GSK3-β in this process has not been previously demonstrated. However, it remains unclear how GSK3-β mediates its effect on p22phox and there may be other signalling intermediates involved.

The dramatic reduction in p22phox and ROS upon PKC412 treatment would likely have consequences for downstream signalling relevant to AML. Indeed, targeted knockdown of p22phox resulted in a large reduction in the active, phosphorylated STAT5 and one of its target genes Pim-1 (Figure 5). Both STAT5 and Pim-1 are known to be extremely important in AML development and progression [34]. This result indicates a novel role for p22phox in regulating signalling cascades vital to AML progression at the level of the transcriptional activator STAT5. Pim-1 has recently been shown as a downstream effector of aberrant signalling by mutated FLT3 on the ER [53] and is known to regulate critical pathways in AML [20], [34]. We propose a mechanism for the function of p22phox in the mediation of STAT5 signalling in AML in Figure 6, based on our data presented here. p22phox plays a role in maintaining essential signalling pathways in AML and it achieves this through facilitating the production of ROS. Interfering with endogenous ROS levels here by knocking down p22phox is enough to stop STAT5 signalling. Given the importance of STAT5 in AML this highlights the importance of the redox state of the cell in AML, and tumours in general. Also it is worth noting that targeting of p22phox may be of therapeutic benefit given that our data show it to be upstream of these pathways.

## Supporting Information

Figure S1 **H_2_O_2_ reduction in MV4-11 AML cells upon FLT-3 and Nox-inhibition.** Densitometry analysis of data represented in Figure 1. Digital images were analyzed with Meta-Morph software. Bar chart represents mean ± SE and are representative of three independent experiments.
(TIF)

Figure S2 **Mitochondrial ROS levels are unaffected by FLT3-inhibition in MV4-11 AML cells.** Densitometry analysis of data represented in Figure 1. Digital images were analyzed with Meta-Morph software. Bar chart represents mean ± SE and are representative of three independent experiments.
(TIF)

Figure S3 **siRNA mediated reduction of p22phox in MV4-11 AML cells.** Densitometry analysis of data represented in Figure 2. Digital images were analyzed with Meta-Morph software. Bar chart represents mean ± SE and are representative of three independent experiments.
(TIF)

Figure S4 **Ubiquitination of p22phox upon FLT3-inhibition in MV4-11 AML cells.** Immunoprecipitation of ubiquitinated proteins from whole-cell lysates of untreated MV4-11 cells (Unt) and cells treated with PKC412 (250nM) for 8 hours. Nitrocellulose membranes were probed for the presence of p22phox. Values are the mean ± SD and are representative of three independent experiments.
(TIF)
