# Supplementary figures and images for: H2O2 Production Downstream of FLT3 Is Mediated by p22phox in the Endoplasmic Reticulum and Is Required for STAT5 Signalling
Source: PLoS One. 2012 Jul 13;7(7):e34050. doi: 10.1371/journal.pone.0034050 (PMC3396659; doi:10.1371/journal.pone.0034050)

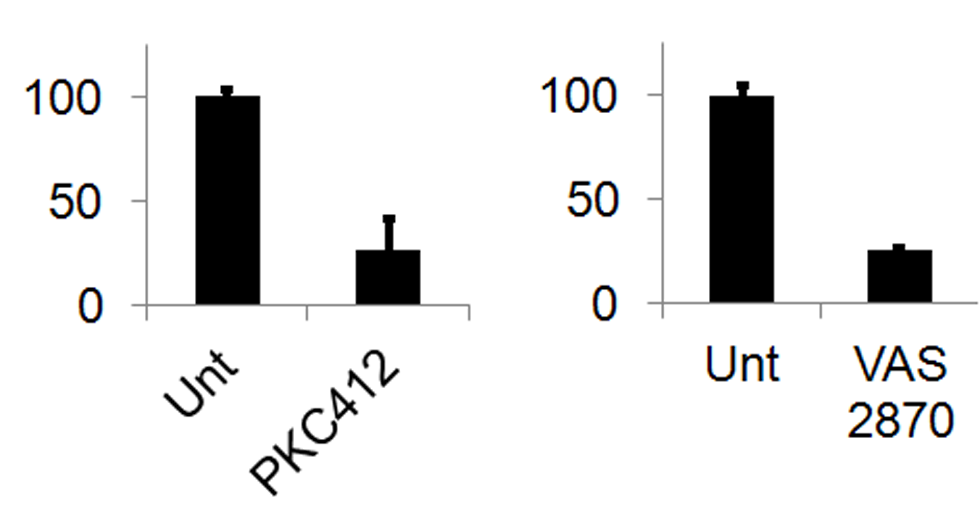

Supplement: Figure S1 — H2O2 reduction in MV4-11 AML cells upon FLT-3 and Nox-inhibition. Densitometry analysis of data represented in Figure 1. Digital images were analyzed with Meta-Morph software. Bar chart represents mean ± SE and are representative of three independent experiments. (TIF) [file pone.0034050.s001.tif]

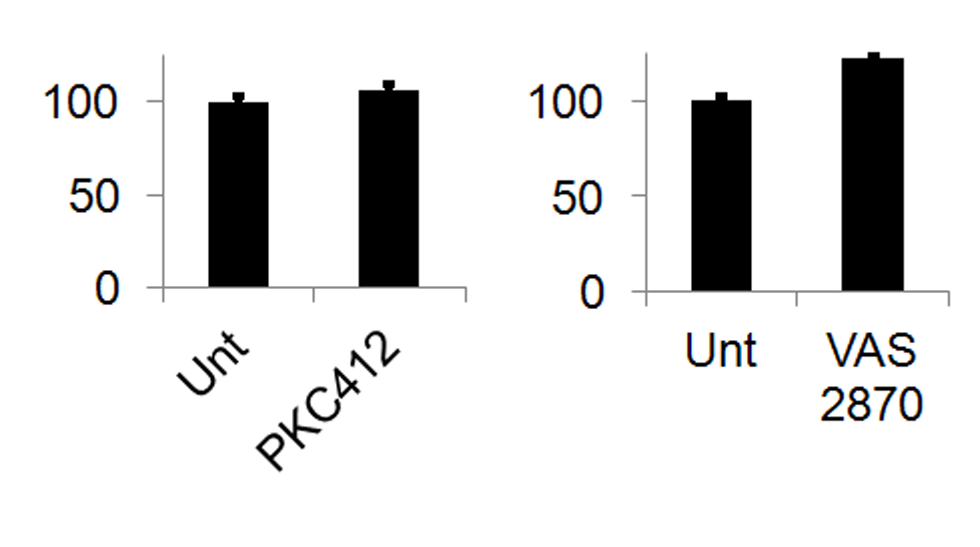

Supplement: Figure S2 — Mitochondrial ROS levels are unaffected by FLT3-inhibition in MV4-11 AML cells. Densitometry analysis of data represented in Figure 1. Digital images were analyzed with Meta-Morph software. Bar chart represents mean ± SE and are representative of three independent experiments. (TIF) [file pone.0034050.s002.tif]

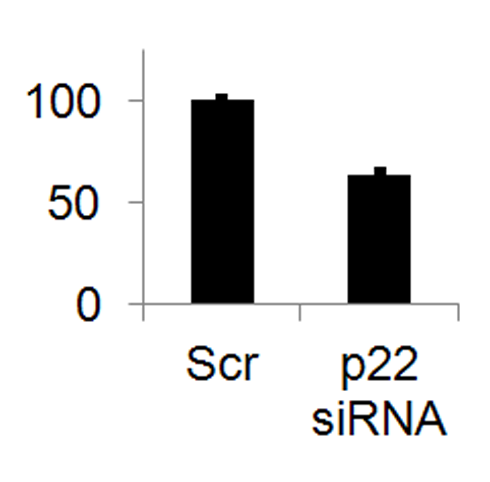

Supplement: Figure S3 — siRNA mediated reduction of p22phox in MV4-11 AML cells. Densitometry analysis of data represented in Figure 2. Digital images were analyzed with Meta-Morph software. Bar chart represents mean ± SE and are representative of three independent experiments. (TIF) [file pone.0034050.s003.tif]

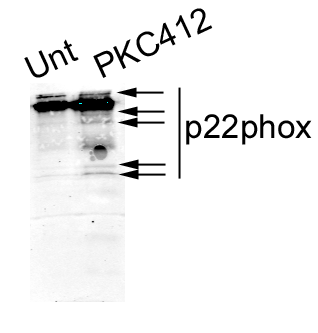

Supplement: Figure S4 — Ubiquitination of p22phox upon FLT3-inhibition in MV4-11 AML cells. Immunoprecipitation of ubiquitinated proteins from whole-cell lysates of untreated MV4-11 cells (Unt) and cells treated with PKC412 (250nM) for 8 hours. Nitrocellulose membranes were probed for the presence of p22phox. Values are the mean ± SD and are representative of three independent experiments. (TIF) [file pone.0034050.s004.tif]
